# Evaluating the Environmental and Biological Safety of Phosphonium-Based Porous Liquid Systems

**DOI:** 10.1021/acsomega.6c09198

**Published:** 2026-10-29

**Authors:** Marcin Wysokowski, Tomasz Rzemieniecki, Łukasz Ławniczak, Kacper Sturma, Adam Gorczyński, Michał Niemczak, Teofil Jesionowski

**Affiliations:** † Faculty of Chemical Technology, Institute of Chemical Technology and Engineering, 49632 Poznan University of Technology, Berdychowo 4, Poznan 61131, Poland; ‡ Faculty of Chemistry, Adam Mickiewicz University in Poznań, Uniwersytetu Poznańskiego 8, Poznań 61614, Poland

## Abstract

Porous liquids have emerged as a promising class of advanced materials that combine permanent porosity with fluidity. They are often proposed as “green” alternatives in separations and catalysis, although their biological safety remains underexplored. Here, we present a comprehensive evaluation of the toxicity of two representative porous liquid systems based on methyltriphenylphosphonium bromide ([P_1_Ph_3_]­[Br]) and trihexyltetradecylphosphonium bis­(trifluoromethylsulfonyl)­amide ([P_66614_]­[NTf_2_]), combined with glycerol and ZIF-8, across a spectrum of biological models. Acute toxicity was assessed in microorganisms (*Escherichia coli*), aquatic organisms (*Daphnia magna*, *Raphidocelis subcapitata*, *Artemia franciscana*), plants (tomato and oat), and human dermal fibroblasts. While both materials exhibited low cytotoxicity toward human dermal fibroblasts (IC_50_ > 0.5 mM), notable toxicity emerged in aquatic organisms and bacteria, especially for aromatic [P_1_Ph_3_]­[Br]-based systems (EC_50_ = 1–10 mg dm^–3^). The studied multicomponent formulations disrupted microbial growth and inhibited germination even at low concentrations, revealing unexpected phytotoxicity. These contrasting outcomes (limited toxicity toward HDF cells, notable ecotoxicity) highlight the hidden complexity of structure-toxicity relationships in porous liquids. Our findings call for a shift from assumption to evidence in the development of so-called “green” solvents, as functionality must go hand in hand with environmental foresight.

## Graphical abstract

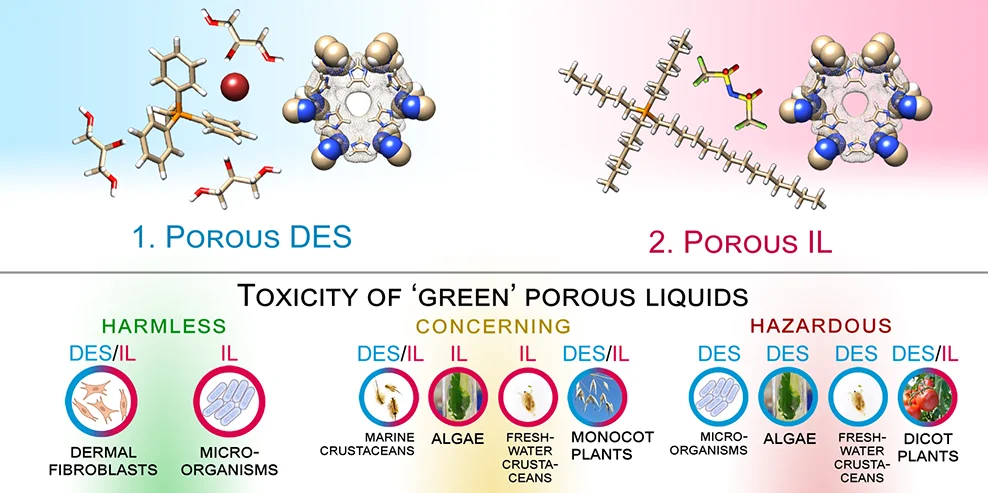

## Introduction

Porous liquids (PLs) are an emerging class of materials that combine the permanent porosity of solids with the fluidity of liquids.^1–3^ This enables novel applications in gas storage,^4^ separation,^5–7^ and catalysis.^8–10^ Their compositions range from discrete molecular cages to colloidal particles stabilized by bulky molecules or electrostatic forces. A unifying design principle across all PLs is size-exclusion adsorption: molecules which are small enough to enter the internal pores are selectively adsorbed, while larger solvent components are excluded, preserving access to porosity in the liquid phase.^3^ Despite their promise, many early porous liquids (particularly those based on molecular cages or designer ionic liquids) faced practical challenges, including complex or costly synthesis routes, limited thermal stability, or the need for structural functionalization to prevent aggregation.^9,11^ These issues triggered an interest in more accessible and sustainable modular systems. Deep eutectic solvents (DESs), formed by combining hydrogen bond donors (HBDs) and acceptors (HBAs) to create eutectic mixtures, emerged as attractive candidates. DESs exhibit several advantages such as their low volatility, compositional flexibility, and ease of preparation from widely available components.^12–17^ Building on this concept, Avila et al.^18^ and Corsini et al.^19^ recently proposed the use of phosphonium-based DES to generate porous liquids by dispersing ZIF-8, a metal–organic framework, into a glycerol-containing eutectic mixture. Their formulation was shown to maintain suspension stability and porosity over several months, with enhanced gas absorption properties and electrochemical compatibility – features that reinforce the perceived green credentials of porous DESs.^18,19^ This approach represents a smart and elegant evolution in porous liquid design and may indeed mark a potential milestone in the development of functional, tunable, and accessible porous liquids. However, while the structural and physicochemical performance of these systems is well demonstrated, less emphasis has been placed on the toxicological properties of the used components. Recent studies have employed methyltriphenylphosphonium quaternary salt to form deep eutectic mixtures, yet the environmental and biological profiles of these salts raise significant concerns regarding their toxicity. Phosphonium-based ionic liquids (ILs) are stable and persistent in aquatic and terrestrial environments, posing potential long-term ecological risks.

To date, this concern has remained largely theoretical in the context of porous DESs. Yet it echoes a broader pattern observed during the early development of ILs, as initial claims of environmental benignity were later contradicted by systematic toxicity studies. Over the past two decades, extensive toxicological studies have dispelled the early assumption that ILs are inherently environmentally benign.^20^ A comparable evaluation gap exists for DESs. DESs are frequently classified as “green” on the basis that one constituent (e.g., choline chloride, a sugar, or an organic acid) is natural or biocompatible; however, this classification typically does not account for the toxicological contribution of the second component, such as certain hydrogen-bond donors or hydrophobic constituents, which may substantially alter the toxicity profile of the resulting mixture.^21^ In type III porous DES-based systems, a third component (the porous solid phase, e.g., a metal–organic framework) introduces an additional variable, as residual metal ions, unreacted linkers, or framework degradation products may themselves contribute to the overall toxicity^22,23^ of the formulation. As with ILs, the environmental compatibility of DESs cannot be inferred from the individual components’ origin or biodegradability alone; it must be assessed empirically, using the physicochemical and biological properties of the complete formulation. Standardized ecotoxicity protocols, such as those recommended by the OECD^20,24^ provide a basis for this assessment and are applied in the present study to the DES-based porous liquid systems described above. To clarify this issue, we assessed the environmental compatibility of two recently reported porous liquid systems (Figure 1) proposed for gas capture: one based on a deep eutectic solvent ([P_1_Ph_3_]­[Br]:Gly:ZIF-8) and the another based on a hydrophobic ionic liquid ([P_66614_]­[NTf_2_]:ZIF-8). We hypothesized that the environmental profile of these systems cannot be inferred solely from their classification as porous DESs or ILs, but must be established through standardized toxicological assessment. We conducted standardized toxicity assays using the complete porous liquids as well as their respective DES and IL components. Tests were performed on a broad range of biological models, including microorganisms (*Escherichia coli*) three aquatic species representing different trophic levels (*Raphidocelis subcapitata*, *Daphnia magna*, *Artemia franciscana*), two edible plant species (*Avena sativa* L. and *Solanum lycopersicum*), and normal human dermal fibroblasts (HDFs).

**Figure 1:**
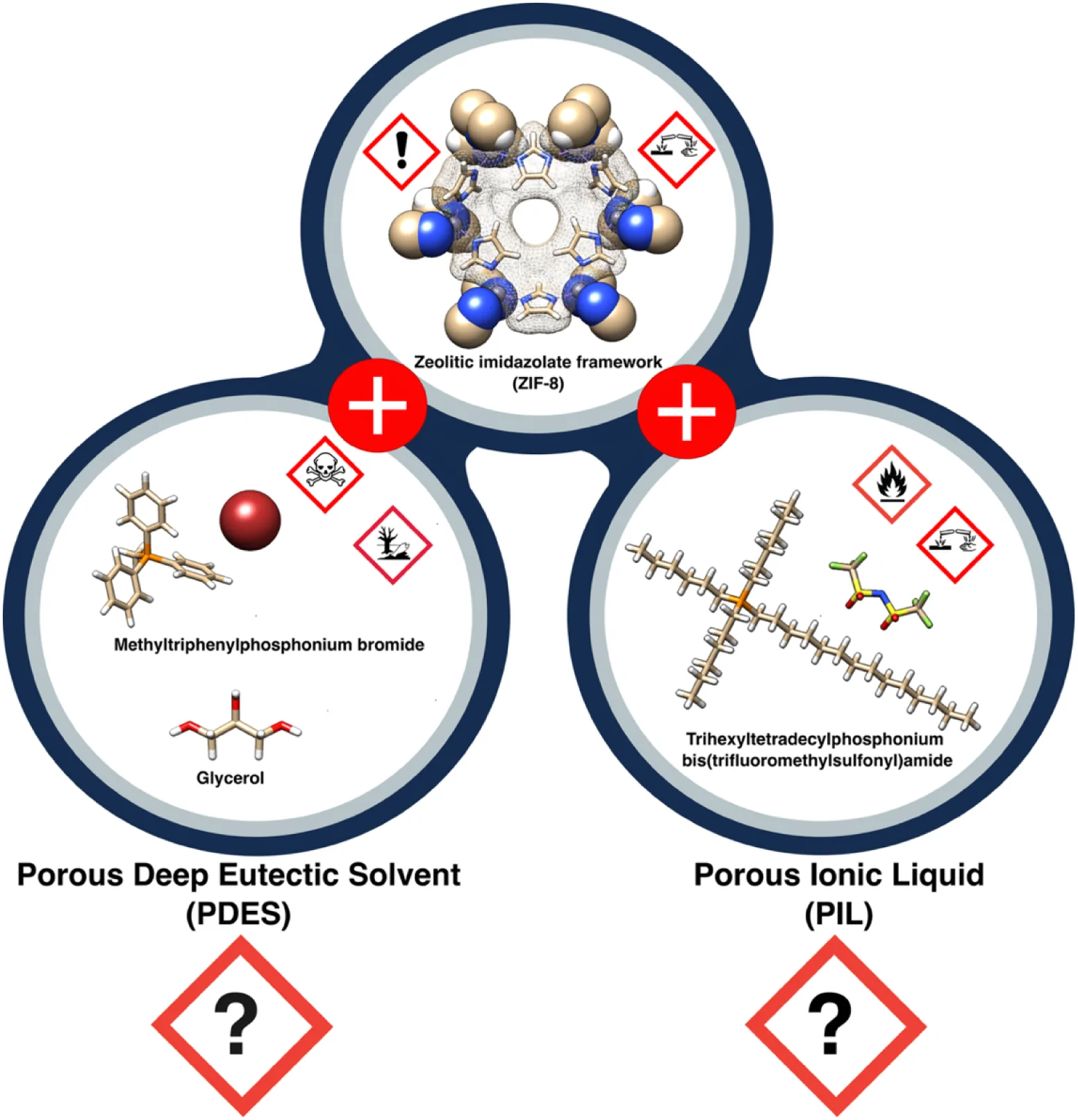
Schematic representation of two porous liquid systems investigated in this study. Each individual component is marked with its respective GHS hazard pictogram to reflect known safety data; note that the toxicity of the final multicomponent systems may differ due to potential synergistic or mitigating effects.

## Experimental Section

### Materials

Zinc acetate dihydrate, 2-methylimidazole, methyltriphenylphosphonium bromide ([P_1_Ph_3_]­[Br]), trihexyltetradecylphosphonium bis­(trifluoromethylsulfonyl)­amide ([P_66614_]­[NTf_2_]), and glycerol were purchased from Sigma-Aldrich and used without further purification.

### Synthesis of ZIF-8

Zeolitic imidazolate framework-8 (ZIF-8) was synthesized using surfactant-free method described by Kim et al.^25^ Zinc acetate dihydrate (1.5 g) was dissolved in 25 mL of distilled water, and 2-methylimidazole (2-mIm, 5.61 g) was dissolved in a separate 25 mL portion of distilled water. The zinc solution was quickly added to the 2-mIm solution under vigorous stirring at room temperature. After 15 min of stirring, the mixture was aged for 24 h without further agitation. The resulting precipitate was recovered by centrifugation and washed five times with distilled water to remove residual reagents.

The diffraction peaks at 2θ values align well with the characteristic reflections of ZIF-8, confirming high phase purity and crystallinity (Figure S1). The absence of additional peaks indicates no impurities or unreacted precursors. The match with literature data validates the effectiveness of the synthesis conditions for producing phase-pure ZIF-8 nanoparticles. The nitrogen adsorption–desorption isotherm (Figure S2a) exhibits a sharp uptake at low relative pressures (P/P_0_ < 0.1), characteristic of a Type I isotherm, indicating a predominantly microporous structure. The pore size distribution (Figure S2b), derived via the DFT method (Figure S2b), reveals prominent peaks around 1.2 and 1.6 nm, consistent with the intrinsic microporosity of ZIF-8. The absence of broader peaks at higher pore widths confirms negligible mesoporosity. Assuming the BET-specific surface area 1226 m^2^/g is in the typical range for ZIF-8.^25–27^ This data supports the formation of a highly microporous, crystalline framework with well-defined pores.

### Preparation of DES

The deep eutectic solvent (DES) was prepared following the method reported by Avila et al.^18,28^ Briefly, a transparent liquid DES, denoted as [P_1_Ph_3_]­[Br]:Glycerol (1:3), was obtained by mixing methyltriphenylphosphonium bromide (6.43 g, 0.018 mol) and glycerol (4.97 g, 0.054 mol) in a 1:3 molar ratio. The components were combined using an EasyMax 102 automatic synthesis workstation (Mettler Toledo) and stirred magnetically at 500 rpm at 358 K for 30 min. After cooling to room temperature, the resulting DES was used in subsequent experiments.

### Preparation of Porous Liquids

The previously prepared DES ([P_1_Ph_3_]­[Br]:Glycerol, 1:3) and [P_66614_]­[NTf_2_] were used as liquid phases for the preparation of porous deep eutectic solvent (PDES) and porous ionic liquid (PIL), respectively. In each case, 5 wt % of ZIF-8 was suspended in the corresponding liquid phase. The mixtures were prepared using an EasyMax 102 automatic synthesis workstation (Mettler Toledo) by stirring at 500 rpm at 338 K for 1 h. The resulting dispersions were homogeneous and stable and were used directly for further characterization and cytotoxicity testing.

### Analysis of Toxicity toward Microorganisms

The toxicity assay was conducted using strains of *Escherichia coli* ATCC 8739, which were initially stored under refrigerated conditions and resuspended using a 50% TS broth (Sigma-Aldrich, Saint Louis, Missouri, USA). After an initial incubation (24 h, 30 °C), the culture was used to prepare experimental samples, which consisted of 1 mL of the bacterial suspension, 1 mL of resazurin stock solution and 14 mL of TS broth.

The toxicity toward the tested microorganisms was analyzed by employing plates with a standard 96 well arrangement. Two plates were prepared: first containing the test samples and biotic control (which included microbial cells and lacked the tested compounds), and the second containing abiotic controls (which included the tested compounds and lacked microbial cells). The test samples were prepared by adding 50 μL of the test substance to row A, followed by 200 μL of the *Escherichia coli* inoculum. Subsequent rows (B–H) were supplemented with 50 μL of 2-fold dilutions of the test substances, prepared according to the serial dilution method, followed by 200 μL of the inoculum. The biotic control was introduced to column 12. The plates were then placed in an incubation room under controlled conditions (30 °C) and in a state of constant motion (using a rotary shaker) for a period of 24 h. The experiments were conducted using 3 independent replicates which were visually inspected after termination of the experiment.

### Analysis of Ecotoxicity

#### Green Algae

The algal growth inhibition tests were performed using the freshwater green microalga *Raphidocelis subcapitata* (SAG 61.81), supplied by the Culture Collection of Algae at Göttingen University (SAG), which is an OECD-recommended culture collection. The experimental procedure followed the principles of OECD Guideline 201. Prior to the toxicity experiments, the algae were maintained in sterile BASAL medium. A stock inoculum was transferred to the test medium and preconditioned for 4 days under the same environmental conditions as those applied during the subsequent experiments. During this period, the culture was continuously aerated and exposed to white LED illumination at 5000 ± 1000 lx. Following this acclimation period, the inoculum was diluted with BASAL medium until an absorbance of 0.20–0.30 was obtained when measured using a 10 cm optical path. For the range-finding experiments, 19 mL of the prepared algal suspension was combined with 1 mL of the respective test compound solution prepared in BASAL medium. For the control samples, 1 mL of BASAL medium was added instead. This procedure yielded final test concentrations of 0.1, 1, 10, 100, and 1000 mg/L. Three independent replicates were prepared for each concentration and for the control. Algal growth was monitored by measuring the optical density at 683 nm (OD683) using a 10 cm optical path. Measurements were performed immediately before exposure and after 72 h of incubation. Throughout the experiment, the test cultures were maintained at 25 °C under continuous illumination and mixing. To minimize the influence of positional effects, the test vessels were randomized and their positions were changed once per day. The rate of inhibition of algal growth was subsequently determined from the measured algal concentrations according to the following equation:

$$\mu =\frac{\ln\left({\mathrm{OD}}_{t_{2}}\right)-\ln\left({\mathrm{OD}}_{t_{1}}\right)}{t_{2}-t_{1}}$$

$OD_{t_{2}}$ and $OD_{t_{1}}$ are the absorbance of the tested solution at day *t*_1_ and *t*_2_ in the experiment, respectively. Subsequently, growth rate inhibition was determined in relation to the control mean:

$$\%\mathrm{inhibition}=100\times \left(1-\frac{\mu_{\mathrm{sample}}}{\mu_{\mathrm{control}}}\right)$$

The relationship between the biological response and the concentration of each tested compound was subsequently analyzed graphically, allowing the approximate EC_50_ range to be established.

#### Toxicity toward Crustaceans

The toxic effects of the tested esterquats were evaluated using two crustacean species, *Artemia franciscana* and *Daphnia magna*, with EC_50_ values determined from the obtained responses. For *Daphnia magna*, the experiments were conducted using the DaphtoxkitF assay (MicroBioTests Inc., Ghent, Belgium), following a procedure consistent with OECD Guideline 202 and ISO 6341. The toxicity assessment involving *Artemia franciscana* was performed with the Artoxkit assay (MicroBioTests Inc., Ghent, Belgium), according to a protocol based on the ASTM E1440-91 standard.

#### Artemia franciscana

For the assessment involving the marine crustacean, the experiment was preceded by the preparation and hatching of *Artemia franciscana* cysts. Briefly, 50 mg of cysts were placed in Petri dishes containing 10 mL of artificial seawater adjusted to a salinity of 35 ppt. The seawater served both as the culture medium for organism development and as the matrix used to prepare the test solutions. The cysts were incubated for 30 h at 25 °C under illumination of 6000–10,000 lx. Two hours before transferring the organisms to the multiwell plates, 20 mg of spirulina was supplied to the developing *Artemia* culture. The artificial seawater was additionally aerated for 1 h immediately before the toxicity assay. For each test concentration and the control, 1 mL of the corresponding test solution or artificial seawater, respectively, was dispensed into each of four wells in the assigned row. At the beginning of the assay, at least 30 organisms were transferred from the prepared Petri dishes to the first well of each row, representing the control or one of the five selected test concentrations. Subsequently, organisms were transferred from the first well to the remaining three wells of the same row, with 10 individuals introduced into each well. After the organisms had been distributed, the multiwell plates were sealed with Parafilm and covered with a plastic lid. The prepared plates were incubated at 25 °C in the absence of light. The number of immobilized organisms was recorded after 24 and 48 h of exposure. The percentage of immobilization (%Imm) was calculated relative to the initial number of organisms according to the following equation:

$$\%\mathrm{Imm}=100\times \left(1-\frac{\mathrm{No}\;\mathrm{of}\;\mathrm{mobile}\;\mathrm{organisms}}{\mathrm{No}\;\mathrm{of}\;\mathrm{mobile}\;\mathrm{organisms}\;\mathrm{at}\;\mathrm{start}}\right)$$

The concentration–response relationship was subsequently evaluated graphically, based on which the corresponding EC_50_ region was estimated.

#### Daphnia magna

The DaphtoxkitF test plates and ISO Test water were obtained from MicroBioTests Inc. (Ghent, Belgium). Freshwater *Daphnia magna* neonates, aged less than 24 h, were cultured at Poznan University of Technology and used for the toxicity experiments. One hour prior to initiating the assay, the test medium was aerated, while the organisms were supplied with spirulina 2 h before exposure. For each of the control and five selected test concentrations, 10 mL of the respective test solution was dispensed into each of the five wells assigned to a given row of the DaphtoxkitF plate. At least 20 *Daphnia* were then transferred to the first well of each row corresponding to the control or a selected concentration. Five individuals were subsequently taken from this initial well and transferred to each of the four remaining wells in the same row. Following the distribution of the organisms, the plates were sealed with Parafilm and covered with a plastic lid. The prepared test systems were maintained at 20 °C under dark conditions throughout the exposure period. The percentage of immobilized organisms was determined, and the EC_50_ values were estimated using the same approach as described for *Artemia franciscana*.

### Analysis of Phytotoxicity

Phytotoxicity of the studied compounds was evaluated using a seed germination assay. The model plants selected for the study included common edible plants, namely oat (*Avena sativa* L.) and tomato (*Solanum lycopersicum* L.). Commercially available seeds of both plants were purchased from W. Legutko (Poland). The experiments were conducted with the use of standard kits for phytotoxicy testing (Tigret, Poland) and a commercial soil substrate (SIA SILUFLORA, Latvia). To this end, 50 g of soil and 7 mL of the studied compound solution or pure water (controls) were combined, homogenized and introduced into one chamber of the kit, which allowed to obtain the values corresponding to 0.1, 1.0, and 10.0 g of the tested substance per 1 kg of soil. Then, 10 seeds of the selected model plant were placed evenly into each experimental trial and the kits were subsequently left in a dark room for 7 days. Following this period, the germinated seeds were removed from the kits and the root and shoot length of the emerged seedlings was measured. Each result is an average value calculated based on measurements carried out for 10 independent replicates and the values were compared to control samples in order to establish any inhibitory effects.

### Cytotoxicity Assessment

Cytotoxicity testing was carried out in accordance with ISO 10993-5:2009 guidelines using normal human dermal fibroblasts (HDF, ScienCell). Cells were cultured in FM Fibroblast Medium (ScienCell, Lot No. 37980) supplemented with Fibroblast Growth Supplement (FGS, ScienCell, Lot No. 39767), 2% fetal bovine serum (FBS, Gibco, Lot No. B2802149RP), and 1% penicillin–streptomycin (Biowest, Lot No. MS028A). Cells were maintained under standard conditions (37 °C, 5% CO_2_, humidified atmosphere) and passaged according to the supplier’s protocol.

For [P_1_Ph_3_]­[Br]; [P_1_Ph_3_]­[Br]:Gly; and [P_1_Ph_3_]­[Br]:Gly:ZIF-8, stock solutions were prepared in fully supplemented FM Medium (phenol red-free) and diluted to yield test concentrations of 0, 0.1, 1, 5, 10, 50, and 100 mM (referring to the active component).

For [P_66614_]­[NTf_2_] and [P_66614_]­[NTf_2_]:ZIF-8, stock solutions were prepared in dimethyl sulfoxide (DMSO). Final concentrations tested were 0, 1, and 5 mM for compound 4, and 0, 1, 5, and 7 mM for compound 5. DMSO was added to cell cultures at a final concentration of 0.5% (v/v), which is the highest concentration shown not to exert cytotoxic effects on fibroblasts. Due to solubility limitations in DMSO, higher concentrations could not be tested for these compounds.

Cytotoxicity was assessed using the MTT assay based on direct contact exposure. Serial dilutions of the test compounds were prepared in fully supplemented FM Medium, and HDF cells were exposed to each concentration for 24 h. Following incubation, cell viability was assessed by the reduction of MTT [3-(4,5-dimethylthiazol-2-yl)-2,5-diphenyl tetrazolium bromide] to formazan. Absorbance was measured at 570 nm using a microplate reader. IC_50_ values (the concentration reducing cell viability to 50%) were calculated from dose–response curves using nonlinear regression. Three experimental controls were used: (i) Negative control (NC): cells cultured in complete FM Medium without any test compound; (ii) Positive control (PC): cells treated with 0.01% Triton X-100 in FM Medium; (iii) Blank (B): supplemented FM Medium without cells.

### Statistical Analysis

To evaluate whether the observed phytotoxic effects differed significantly among the investigated treatments, statistical analyses were conducted using Origin 2022. Differences between groups were initially assessed by one-way analysis of variance (ANOVA), with statistical significance defined at *p* < 0.05. When significant differences were detected, Tukey’s posthoc procedure was applied to determine the honestly significant difference (HSD) at the 5% significance level. In the graphical presentation of the results, treatments marked with different lowercase letters were considered significantly different from one another.

## Results and Discussion

In case of multicomponent systems, such as the materials evaluated in the framework of this study, the toxic effect may result from the presence of a single substance, several compounds or specific interactions which occur between all elements present in the materials. For this reason, we conducted environmental toxicity studies using porous liquid systems as well as on their IL/DES initial components. We started with pure phosphonium salts, as these compounds were assumed to exhibit the highest toxicity and compared the results with samples which included additional components to observe potential synergy.

### Toxicity toward Microorganisms

The initial tests were focused on the assessment of toxicity toward microorganisms, owing to the fact that they are responsible for biodegradation processes and serve as the first line of defense in case of environmental pollution. The bacterial species selected for the tests (*E. coli*) is widely spread and commonly present in every environmental niche. The resazurin assay allows for rapid evaluation of potential toxic effects – under standard conditions (i.e., lack of toxicity) microbial cells secrete enzymes which react with resazurin and result in a pink color, whereas in cases when the microbial growth is inhibited by the presence of toxic substances the solution retains the original dark blue color of resazurin. Results of this initial stage of studies are presented in Figure 2.

**Figure 2:**
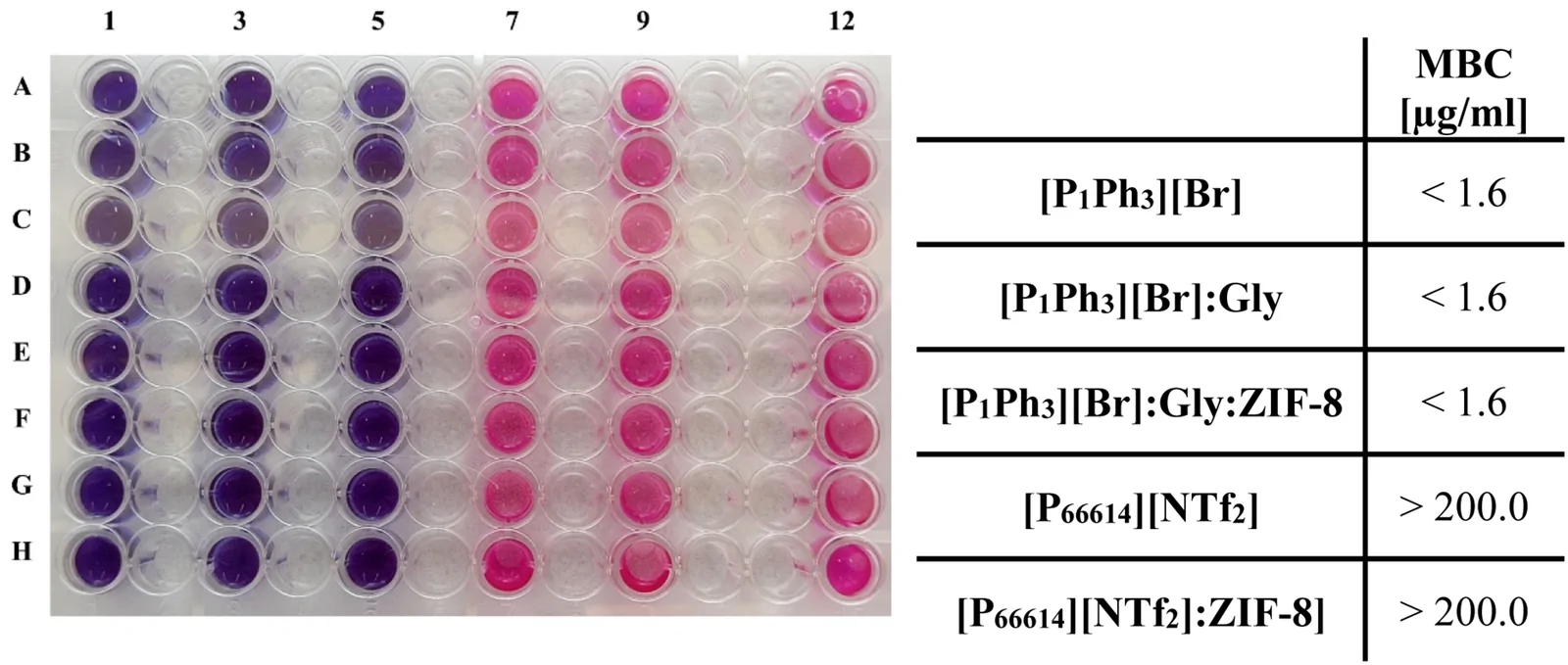
Evaluation of toxicity of the studied systems toward *E. coli* cells and MBC values. Concentrations in respective rows: A) 200.0 μg/mL, B) 100.0 μg/mL, C) 50.0 μg/mL, D) 25.0 μg/mL, E) 12.5 μg/mL, F) 6.3 μg/mL, G) 3.1 μg/mL, H) 1.6 μg/mL. Respective columns include: 1) [P_1_Ph_3_]­[Br], 3) [P_1_Ph_3_]­[Br]:Gly, 5) [P_1_Ph_3_]­[Br]:Gly:ZIF-8, 7) [P_66614_]­[NTf_2_], 9) [P_66614_]­[NTf_2_]:ZIF-8], 12) biotic control.

The obtained results indicated that [P_1_Ph_3_]­[Br] and systems which included this component ([P_1_Ph_3_]­[Br]:Gly and [P_1_Ph_3_]­[Br]:Gly:ZIF-8) were characterized by toxic influence throughout the whole studied range of concentrations. These samples completely inhibited the growth of *E. coli* even at the lowest studied concentrations, which is evidenced by the lack of color shift from blue to pink. In contrast, both [P_66614_]­[NTf_2_] and [P_66614_]­[NTf_2_]:ZIF-8] did not exhibit toxicity at any of the studied concentration thresholds. This notable difference in terms of performance between both groups may result from the character of the alkyl substituents attached to the phosphonium cation (aromatic vs aliphatic), the presence of specific anions (bromide vs bis­(trifluoromethylsulfonyl)­amide which interacts with the cation, or both.

It should be highlighted that the toxic effects of triphenylphosphonium cations (TPP^+^) toward microorganisms have been reported previously in the literature.^29^ Enhanced antimicrobial activity of TPP homologues was observed by Nunes et al. with minimal inhibitory concentrations as low as 0.25 μg/mL against *Staphylococcus aureus*, *Candida albicans*, and *E. coli*.^30^ The Authors highlighted that the enhanced activity of TPP^+^ may result from enhanced delocalization and stabilization of the π-electrons in aromatic rings present in TPP^+^ or facilitated permeation such derivatives through negatively charged bacterial membranes. These findings correspond well with the results presented in the framework of this study. In comparison, the presence of alkyl groups in [P_66614_]­[NTf_2_] or increased stability of the ionic pair due to the presence of a different ion may limit its toxicity and thus result in lower environmental impact.

### Analysis of Ecotoxicity

Subsequent tests were performed on three model species of aquatic organisms belonging to different trophic levels: freshwater green algae (*R. subcapitata*), freshwater crustaceans (*D. magna*), and marine crustaceans (*A. franciscana*). The results of the study are presented in Figure 3.

**Figure 3:**
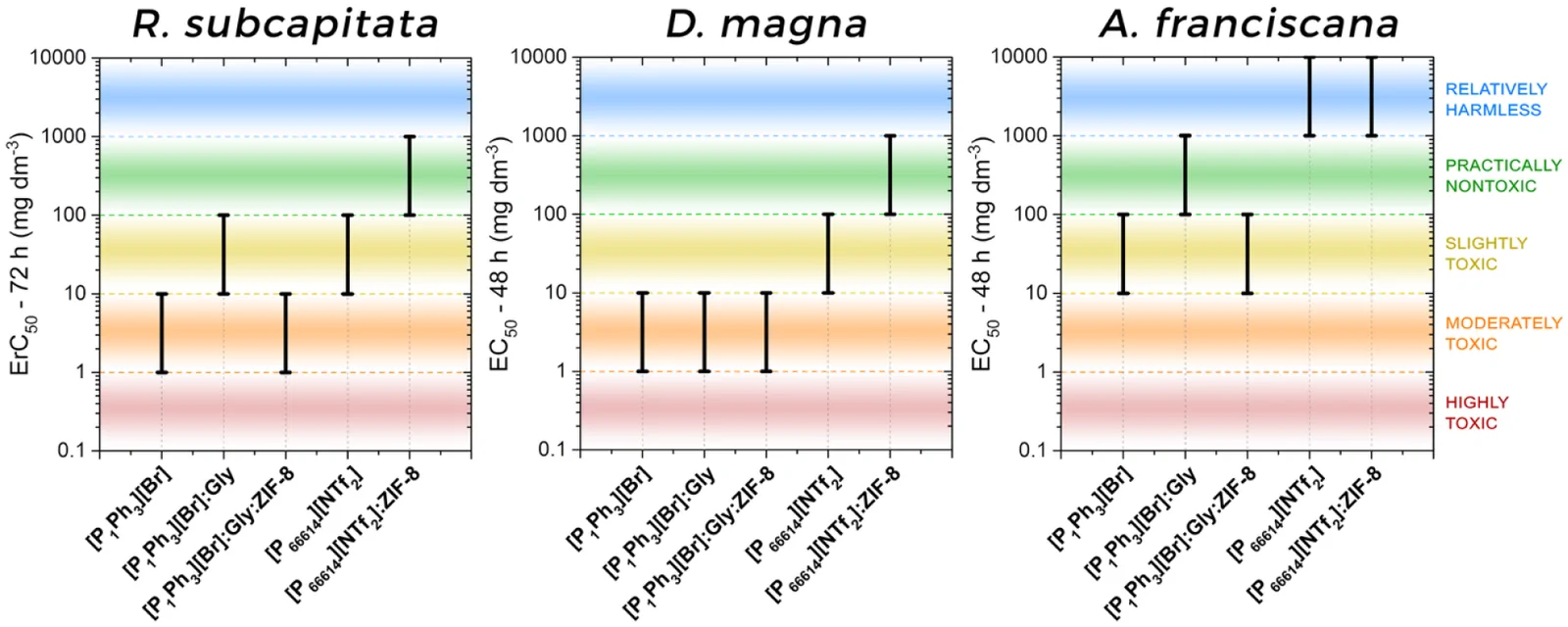
Ecotoxicity classification of porous liquids and their corresponding IL/DES solvents based on toxicity to aquatic organisms.^31^

The results revealed that methyltriphenylphosphonium bromide ([P_1_Ph_3_]­[Br]), a component of DES and the solvent of the tested porous liquid [P_1_Ph_3_]­[Br]:Gly:ZIF-8, exhibits significant acute toxicity toward *R. subcapitata* algae and *D. magna* crustaceans. The EC_50_ value was in the range from 1 to 10 mg dm^–3^, which corresponds to toxicity class 2 according to the GHS (“moderately toxic”^31^). Saltwater crustaceans (*A. franciscana*) exhibited lower, but still noticeable susceptibility to this compound (EC_50_ in the range of 10–100 mg dm^–3^). Due to nontoxicity of glycerol and its high content in the obtained DES, [P_1_Ph_3_]­[Br]:Gly system had similar or lower acute toxicity toward test organisms compared to pure [P_1_Ph_3_]­[Br]. It should be noted that ZIF-8 is toxic to aquatic organisms;^32^ therefore, the addition of the porous material caused an increase in acute toxicity toward *R. subcapitata* and *A. franciscana* compared to [P_1_Ph_3_]­[Br]:Gly. Consequently, the toxicity of the obtained porous DESs was found to be comparable to that of the parent quaternary phosphonium salt ([P_1_Ph_3_]­[Br]). Therefore, the [P_1_Ph_3_]­[Br]:Gly:ZIF-8 system cannot be considered harmless to aquatic organisms and does not exhibit limited environmental impact. This finding challenges the commonly held assumption that DESs, including DES-based porous liquids, are inherently nontoxic and environmentally benign.

Interestingly, the proposed IL ([P_66614_]­[NTf_2_]) and its porous liquid ([P_66614_]­[NTf_2_]:ZIF-8]) were characterized by a significantly lower level of acute toxicity to the tested organisms compared to their [P_1_Ph_3_]­[Br]-based counterparts. Only slight acute toxicity was observed toward freshwater organisms (*R. subcapitata*, *D. magna*) in the case of pure IL, while the EC_50_ of porous liquid exceeded 100 mg dm^–3^, which corresponds to relative lack of acute toxicity. These systems were characterized by even lower toxicity to saltwater organisms (*A. franciscana*, EC_50_ in the range of 1000–10000 mg dm^–3^). Although the observed low acute toxicity likely results from the limited bioavailability of the water-insoluble ionic liquid, this may lead to the misleading conclusion that the proposed IL-based porous liquids are environmentally safe and pose little risk across different species of aquatic organisms. Nevertheless, given the pronounced hydrophobicity of [P_66614_]­[NTf_2_], it is important to highlight the plausible risk of its bioaccumulation and the potential for chronic toxic effects on organisms across multiple trophic levels. Furthermore, according to the current state of knowledge regarding toxicity of ILs, systems containing fluorinated anions (including [NTf_2_]) or cations containing longer alkyl substituents (like [P_66614_]) are significantly more toxic to aquatic ecosystems than their hydrophilic analogues.^33,34^ It should be noted that various reports focused on a direct comparison of toxicity between quaternary ammonium (QASs) and quaternary phosphonium salts (QPSs) indicated that in general, QPSs are slightly more toxic than QASs.^35^ Considering *R. subcapitata* and *D. magna*, the differences in EC_50_ values typically do not exceed 100 mg dm^–3^. Nevertheless, this may indicate that cations based on positively charged nitrogen are more promising candidates for designing systems with genuinely low environmental impact.

### Analysis of Phytotoxicity

The next stage of studies focused on evaluation of toxic effects of the studied compounds during the early stages of plant growth using oat (*Avena sativa* L.) and tomato (*Solanum lycopersicum* L.) as model plants. Oat and tomato serve as representative monocot and dicot species, enabling comprehensive evaluation of phytotoxic effects across plant types. The experiment investigated potential toxicity at the stage of germination using shoot and root growth as indicators of phytotoxicity. The results are presented in Figure 4 (exact walues and SD are presented in Tables S1 and S2).

**Figure 4:**
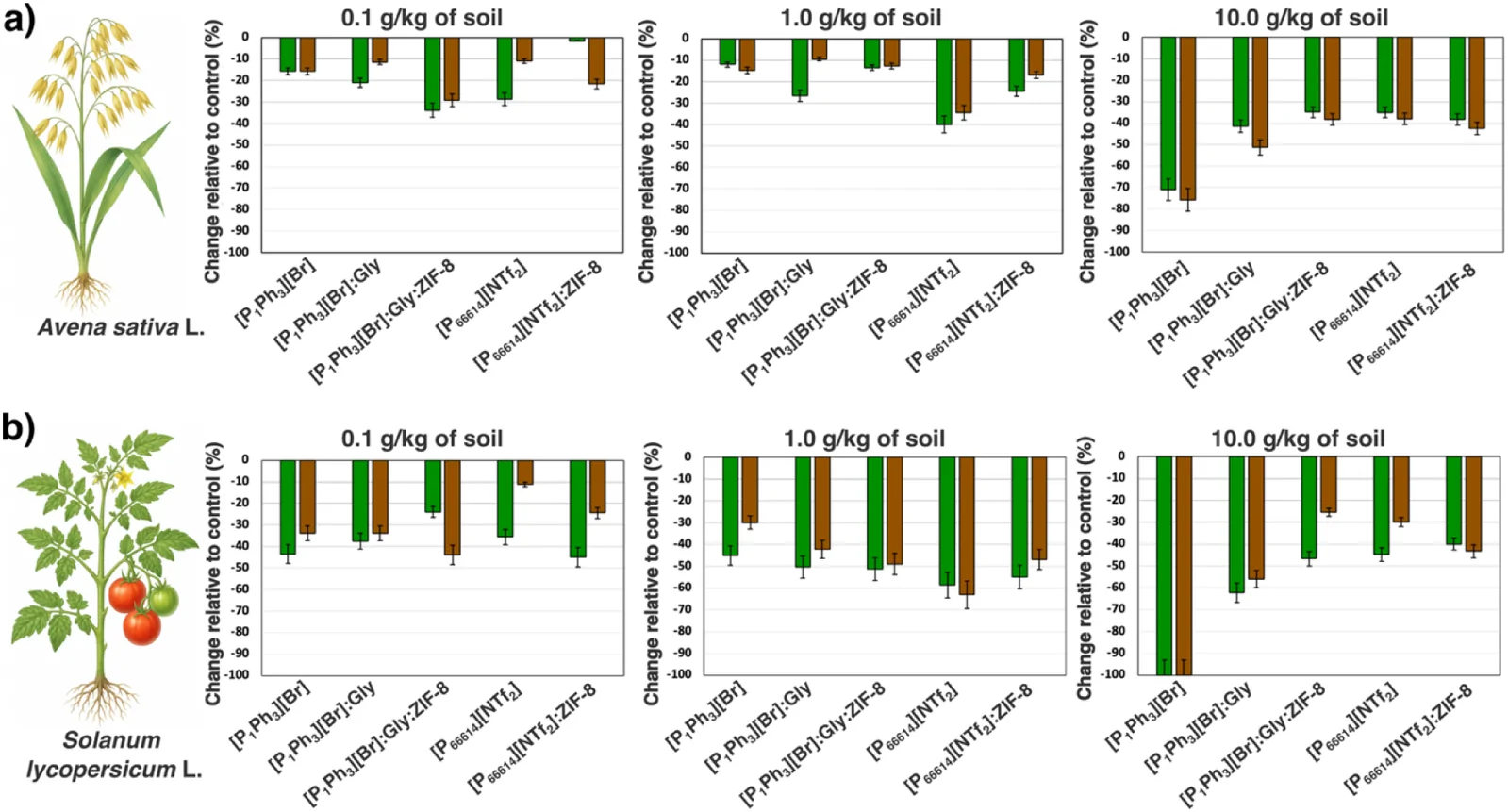
Phytotoxic effects of the studied systems at different concentrations expressed as reduction percentage of shoot (green) or root (brown) length of oat (a) and tomato (b).

The results revealed several noteworthy trends and provided valuable insights into the phytotoxic effect of the studied compounds. First, a distinct difference in terms of susceptibility of the studied plants toward the tested compounds could be observed. Tomato was much more vulnerable and exhibited moderate phytotoxic responses even at the lowest studied concentration (shoot and root reduction in the range of 24–45%), which became more severe as the dose of the potential toxicants increased (30–63% and 26–100%, respectively). In comparison oat was more resistant and showed symptoms of notable phytotoxicity only at the highest dose (overall shoot and root reduction in the range of 1–75%). Second, there was no clear correlation regarding the reduction of shoot vs root. In most cases both parameters suffered from comparable reduction with some exceptions when one was decreased more compared to the other. However, such cases did not carry over to other concentrations. Finally, the phytotoxicity of compounds depended strongly on the studied concentration. Interestingly, at the lowest studied concentration the highest phytotoxic effects were observed for the most complex systems ([P_1_Ph_3_]­[Br]:Gly:ZIF-8 and [P_66614_]­[NTf_2_]:ZIF-8]), reaching a reduction by approximately 30% in oat and by 40% in tomato. In case of the medium dose, the addition of pure [P_66614_]­[NTf_2_] resulted in the most toxic effects (reduction by approximately 40% in oat and by 60% in tomato). Upon exposure of the plants to the highest tested concentration, [P_1_Ph_3_]­[Br] exhibited the most potent phytotoxic effect (reduction by approximately 70% in oat and complete inhibition of growth in case of tomato).

The experiment elucidates several important aspects associated with the presence of the studied compounds in soil, especially in terms of their potential impact on plants. The phytotoxic effects seem to be species specific, and as such they will strongly depend on the type of plant present in a given area. Since tomatoes are dicotyledons and oats are monocotyledons, it is plausible that the first group may be more susceptible to exposure, however this aspect should be studied further using different plant species. While some phytotoxic responses should be expected even in case of resistant plants (such as oat), the extent of possible reduction in seed germination as well as the shoot and root length in seedlings will vary depending on the concentration and type of the contaminant. Although the highest phytotoxicity was observed in case of [P_1_Ph_3_]­[Br], which is consistent with other results, it should be highlighted that the multicomponent systems ([P_1_Ph_3_]­[Br]:Gly:ZIF-8 and [P_66614_]­[NTf_2_]:ZIF-8]) showed signs of negative impact at the lowest studied dose. To this extent plants reacted differently compared to microorganisms, algae and crustaceans, which justifies the need to conduct broad spectrum toxicity studies.

### Cytotoxicity Assessment

Cytotoxicity testing of [P_1_Ph_3_]­[Br] (Figure 5), its formulation with glycerol and final porous eutectic liquid [P_1_Ph_3_]­[Br]:Gly:ZIF-8 revealed a low toxicity toward human dermal fibroblast (HDF) cells, with an IC_50_ values of 0.68 mM, 0.68 mM, and 0.87 mM respectively – well above commonly accepted cytotoxic thresholds.^36^ These findings indicate that the tested porous DES formulations do not elicit significant cell membrane damage or metabolic disruption in fibroblast cells under *in vitro* conditions. One possible explanation for such low cytotoxicity is the relatively low cellular uptake or slow membrane permeability of the phosphonium salt in fibroblasts, potentially due to the bulky aromatic structure^30^ and its interaction with serum proteins in culture medium. Importantly, the MTT assay provides an assessment of cell viability under the tested conditions and does not capture other cellular responses or toxicological effects. Therefore, the results obtained with HDF should not be interpreted as evidence of overall biocompatibility, and future studies should include complementary cellular end points and additional relevant human cell models, including epithelial cells. Interestingly, in contrast to the noncytotoxic profile in mammalian cells, both [P_1_Ph_3_]­[Br] and [P_1_Ph_3_]­[Br]:Gly:ZIF-8 exhibited pronounced toxicity in aquatic species. This discrepancy may arise from species-specific sensitivity, differences in metabolic pathways, or the heightened vulnerability of aquatic organisms to membrane-active agents and ionic compounds in their external environment. Moreover, aquatic species lack the same protective protein and lipid buffering mechanisms present in mammalian cell culture systems, which can lead to increased bioavailability and toxicity.

**Figure 5:**
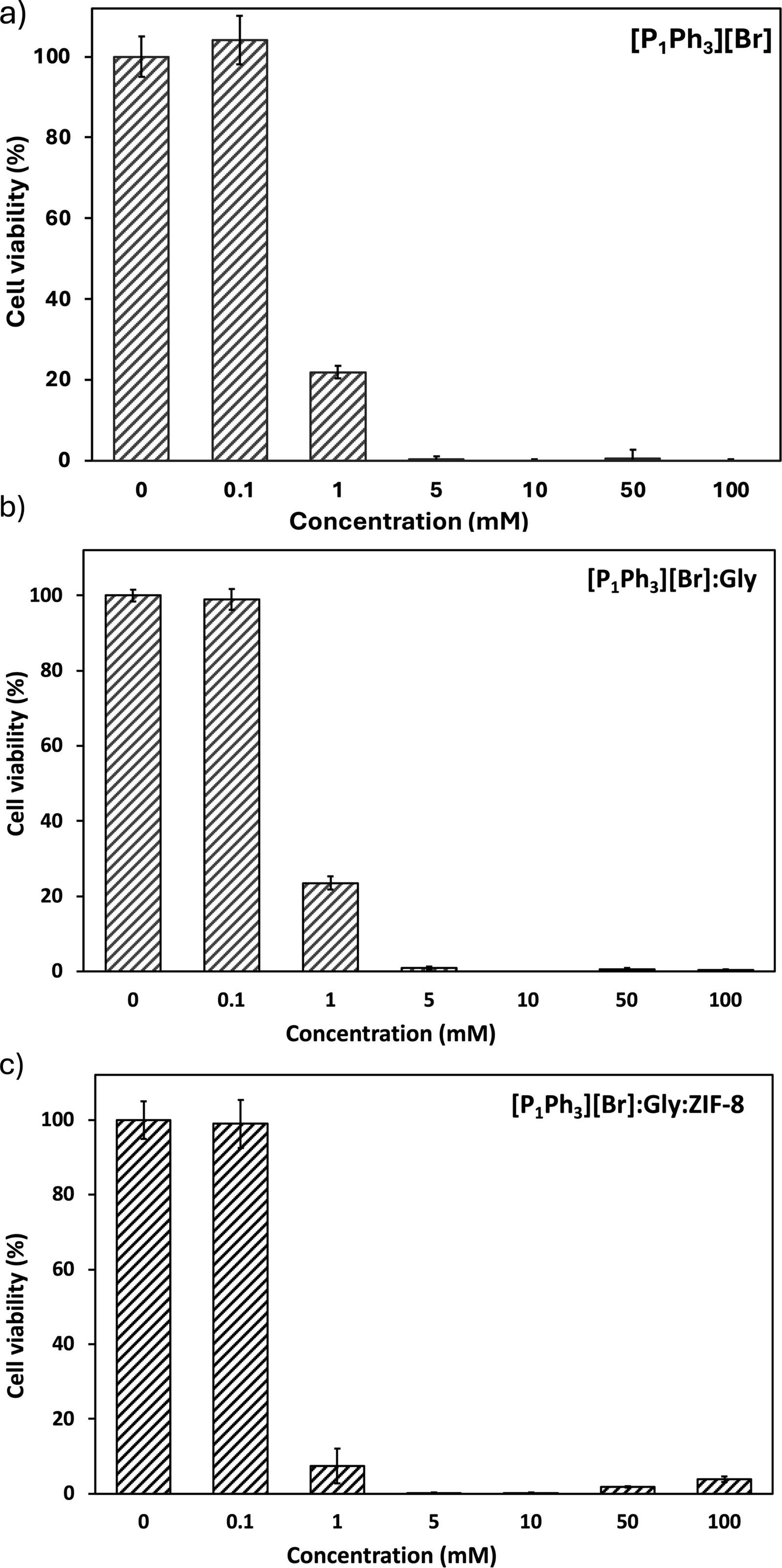
Cell viability of human dermal fibroblasts (HDF) after 24-h exposure to (a) [P_1_Ph_3_]­[Br] and derived formulations (b) [P_1_Ph_3_]­[Br]:Gly and (c) [P_1_Ph_3_]­[Br]:Gly:ZIF-8 assessed via MTT assay.

Notably, [P_66614_]­[NTf_2_] exhibited a noncytotoxic profile in human dermal fibroblasts (Figure 6), consistent with ISO 10993-5 standards.^37^ The corresponding [P_66614_]­[NTf_2_]:ZIF-8 porous liquid composite maintained approximately 70% cell viability at 7 mM, the highest concentration tested due to solubility limitations. While these results suggest favorable cytocompatibility, it is important to emphasize that cytotoxicity is only one aspect of biocompatibility. Thus, although [P_66614_]­[NTf_2_]-based porous liquids show promise for applications with limited direct human exposure, their broader environmental impact should be carefully considered in future development.

**Figure 6:**
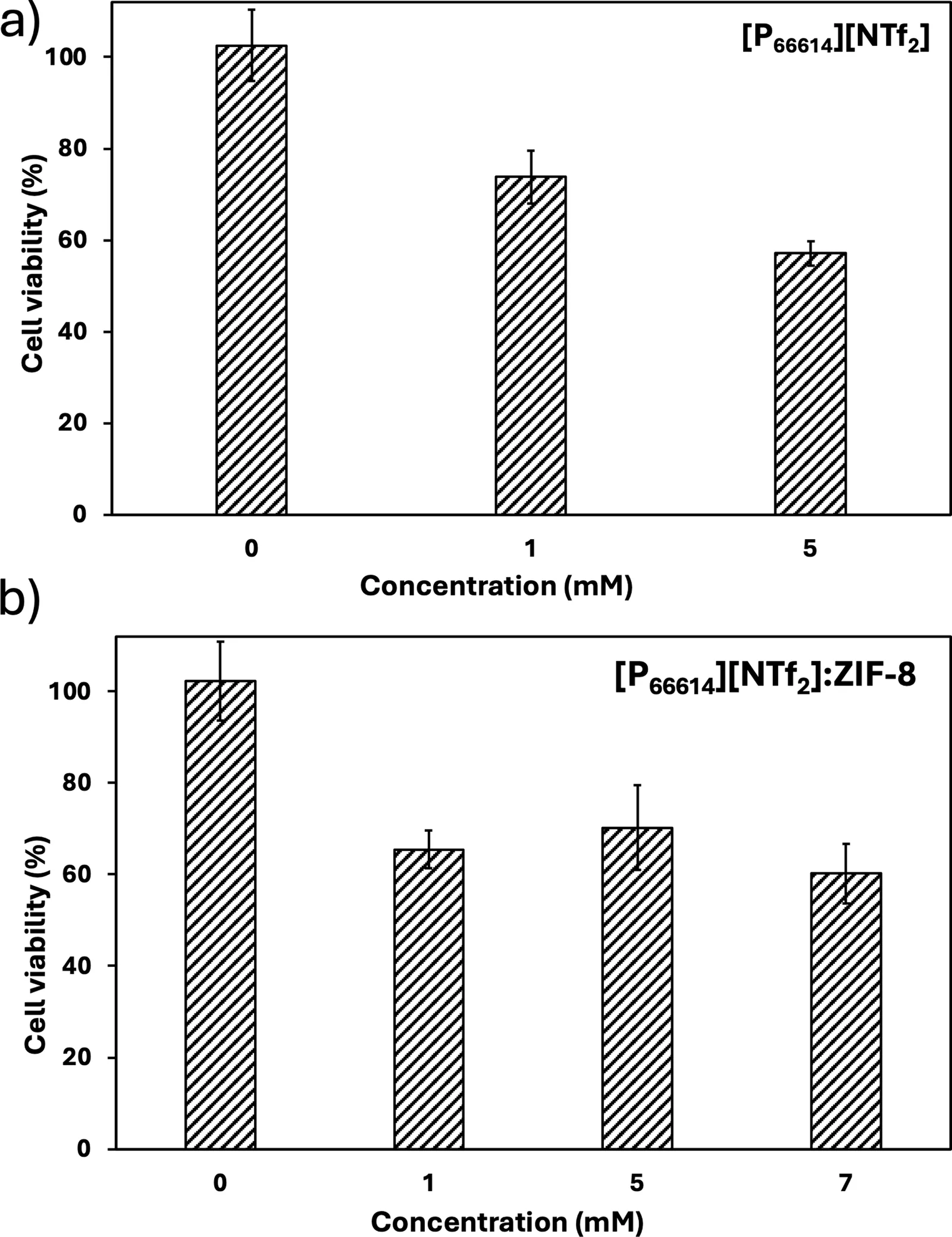
Cell viability of human dermal fibroblasts (HDF) after 24-h exposure to (a) [P_66614_]­[NTf_2_] and derived formulations (b), assessed via MTT assay.

## Conclusions

This study offers a comprehensive assessment of the environmental toxicity and cytotoxicity of two phosphonium-based porous liquid systems, and contributes critical insights into their safety profiles for potential applications at the interface of chemistry, biology, and even materials science.

Initial environmental screening with *E. coli* revealed stark differences in antimicrobial activity between the two phosphonium salt families. [P_1_Ph_3_]­[Br] and its formulations ([P_1_Ph_3_]­[Br]:Gly and [P_1_Ph_3_]­[Br]:Gly:ZIF-8) completely inhibited bacterial growth across all concentrations, likely due to the aromatic cation’s enhanced membrane affinity and π-electron delocalization. In contrast, [P_66614_]­[NTf_2_] and its porous composite showed no antimicrobial activity, which may result from its aliphatic structure and stronger ion pairing with the [NTf_2_]^−^ anion, limiting bioavailability.

Aquatic toxicity tests further differentiated the systems. [P_1_Ph_3_]­[Br]-based compounds exhibited acute toxicity toward *R. subcapitata* and *D. magna* (GHS class 2), with ZIF-8 increasing toxicity, particularly for *R. subcapitata* and *A. franciscana*. In contrast, [P_66614_]­[NTf_2_] and its ZIF-8 composite showed significantly lower toxicity across all tested aquatic organisms tested. However, their hydrophobicity and the environmental persistence of fluorinated anions raise concerns regarding long-term bioaccumulation and chronic effects.

Phytotoxicity testing revealed species-specific sensitivities: tomato (a dicot) exhibited substantial shoot and root inhibition even at low concentrations, while oat (a monocot) was more tolerant but still affected at higher doses. Notably, the multicomponent systems induced phytotoxic responses even at the lowest tested concentrations, in contrast to their performance in microbial and aquatic assays. These results underline the importance of including plant-based models in environmental safety assessments.

Finally, cytotoxicity evaluation using human dermal fibroblasts showed low toxicity across all tested systems. IC_50_ values for [P_1_Ph_3_]­[Br], [P_1_Ph_3_]­[Br]:Gly, and [P_1_Ph_3_]­[Br]:Gly:ZIF-8 were well above commonly accepted thresholds, indicating no significant membrane damage or metabolic disruption under *in vitro* conditions. The discrepancy between low mammalian cytotoxicity and high aquatic toxicity highlights species-specific sensitivities and the protective role of serum proteins and lipid buffering in mammalian systems. This reinforces the need for comprehensive, multimodel testing when assessing the environmental and biological safety of novel functional materials.

The design of porous eutectic solvents assuredly represents a promising approach to creating functional materials with tunable porosity – an innovation that undoubtedly merits exploration on a broader scale, yet the environmental hazards posed by both [P_1_Ph_3_]­[Br]- and [P_66614_]­[NTf_2_]-based systems, particularly under aquatic exposure conditions, cannot be overlooked. Although cytotoxicity in mammalian cells was limited, future work must critically balance the promise of this platform with the ecotoxicological concerns raised here. Future research should prioritize the rational design of porous liquids with minimized inherent toxicity, guided by structure–property relationships and supported by experimental validation. Particular emphasis should be placed on the incorporation of biobased feedstocks and naturally derived compounds, applied not only in the formulation of the liquid/solvent phase but also in the engineering of the porous scaffold itself. However, even when such sustainable design strategies are implemented, these systems must be subjected to comprehensive ecotoxicological evaluation, encompassing multiple trophic levels in both aquatic and terrestrial environments. Our findings indicate that the current state-of-art suffers from several shortcommings. In consequence, future studies should focus on addressing crucial end points such as acute and chronic toxicity, bioaccumulation potential, and long-term ecological persistence. Only through such systematic assessment can these materials be reliably classified as environmentally benign and aligned with the principles of green chemistry. A deeper understanding of species-specific responses and long-term environmental effects will be essential for guiding truly sustainable applications of porous liquid materials.

## Supplementary Material



## Notes

All experimental procedures, analytical methods, and characterization details (PDF)
