# Supplementary material for: Evaluating the Environmental and Biological Safety of Phosphonium-Based Porous Liquid Systems
Source: ACS Omega. 2026 Oct 29;11(44):69143–53. doi: 10.1021/acsomega.6c09198 (PMC13756708; doi:10.1021/acsomega.6c09198)
Supplement: Supplementary file 1 [file ao6c09198_si_001.pdf]

Evaluating the Environmental and Biological Safety of Phosphonium-Based Porous Liquid Systems.

Marcin Wysokowski[*a], Tomasz Rzemieniecki[a], Łukasz Ławniczak[a], Kacper Sturma[a], Adam Gorczyński[b], Michał Niemczak[a], and Teofil Jesionowski[a]*

[a] Faculty of Chemical Technology, Institute of Chemical Technology and Engineering, Poznan University of Technology, Berdychowo 4, 60965 Poznan, Poland,

[b] Faculty of Chemistry, Adam Mickiewicz University in Poznań, Uniwersytetu Poznańskiego 8, Poznań 61614, Poland

Contents:

Experimental (materials and methods)

ZIF-8 characterization results

Microbial toxicity results

Table S1. Phytotoxic effects of the studied compounds towards oat (*Avena sativa* L.)

CONTROL SAMPLE

Average shoot length [cm]	12.65 ± 1.66
Average root length [cm]	9.89 ± 2.61

TREATED SAMPLES

**Studied compounds / dose**		**0.1 g/kg of soil**	**1.0 g/kg of soil**	**10.0 g/kg of soil**
**[P1Ph3][Br]**	Average shoot length [cm]	10.66 ± 0.89	11.13 ± 1.24	3.67 ± 1.24
	Average root length [cm]	8.33 ± 1.68	8.43 ± 2.87	2.4 ± 2.87
**[P1Ph3][Br]:Gly**	Average shoot length [cm]	9.99 ± 2.93	9.28 ± 1.96	7.41 ± 1.96
	Average root length [cm]	8.76 ± 3.72	8.95 ± 3.27	4.82 ± 3.27
**[P1Ph3][Br]:Gly:ZIF-8**	Average shoot length [cm]	8.37 ± 3.26	10.94 ± 1.79	8.23 ± 1.79
	Average root length [cm]	7.01 ± 3.46	8.63 ± 2.56	6.11 ± 2.56
**[P66614][NTf2]**	Average shoot length [cm]	9.03 ± 2.72	7.6 ± 1.38	8.22 ± 1.38
	Average root length [cm]	8.8 ± 1.74	6.49 ± 2.43	6.13 ± 2.43
**[P66614][NTf2]:ZIF-8]**	Average shoot length [cm]	12.45 ± 1.46	9.55 ± 1.95	7.81 ± 1.95
	Average root length [cm]	7.75 ± 1.05	8.22 ± 1.93	5.69 ± 1.93

Table S2. Phytotoxic effects of the studied compounds towards tomato (*Solanum lycopersicum* L.)

CONTROL SAMPLE

Average shoot length [cm]	9.25 ± 2.25
Average root length [cm]	3.60 ± 1.24

TREATED SAMPLES

**Studied compounds / dose**		**0.1 g/kg of soil**	**1.0 g/kg of soil**	**10.0 g/kg of soil**
**[P1Ph3][Br]**	Average shoot length [cm]	5.22 ± 1.12	5.08 ± 1.62	0 ± 0
	Average root length [cm]	2.38 ± 1.05	2.52 ± 1.23	0 ± 0
**[P1Ph3][Br]:Gly**	Average shoot length [cm]	5.77 ± 1.62	4.59 ± 1.85	3.49 ± 0.88
	Average root length [cm]	2.38 ± 1.4	2.08 ± 1.08	1.58 ± 0.55
**[P1Ph3][Br]:Gly:ZIF-8**	Average shoot length [cm]	7.03 ± 2.2	4.51 ± 0.73	4.93 ± 0.79
	Average root length [cm]	2.02 ± 1.56	1.84 ± 1.02	2.68 ± 1.21
**[P66614][NTf2]**	Average shoot length [cm]	5.96 ± 0.45	3.83 ± 1.5	5.1 ± 1.98
	Average root length [cm]	3.2 ± 1.04	1.33 ± 0.56	2.52 ± 1.94
**[P66614][NTf2]:ZIF-8]**	Average shoot length [cm]	5.09 ± 1.24	4.17 ± 1.49	5.55 ± 1.74
	Average root length [cm]	2.72 ± 1.92	1.91 ± 1.17	2.04 ± 1.48

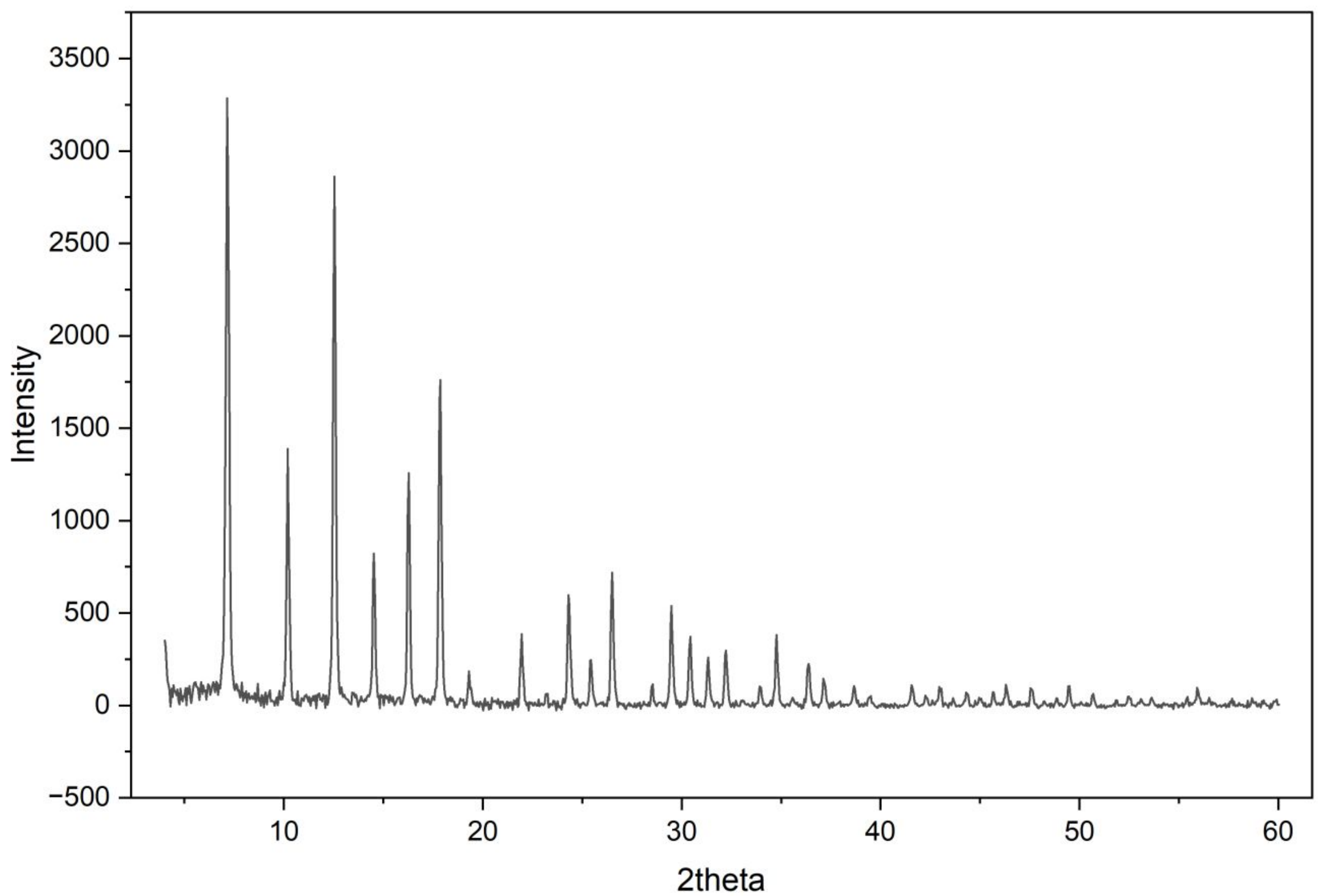

Figure S1. XRD pattern confirming the crystallinity of the synthesized ZIF-8 nanoparticles.

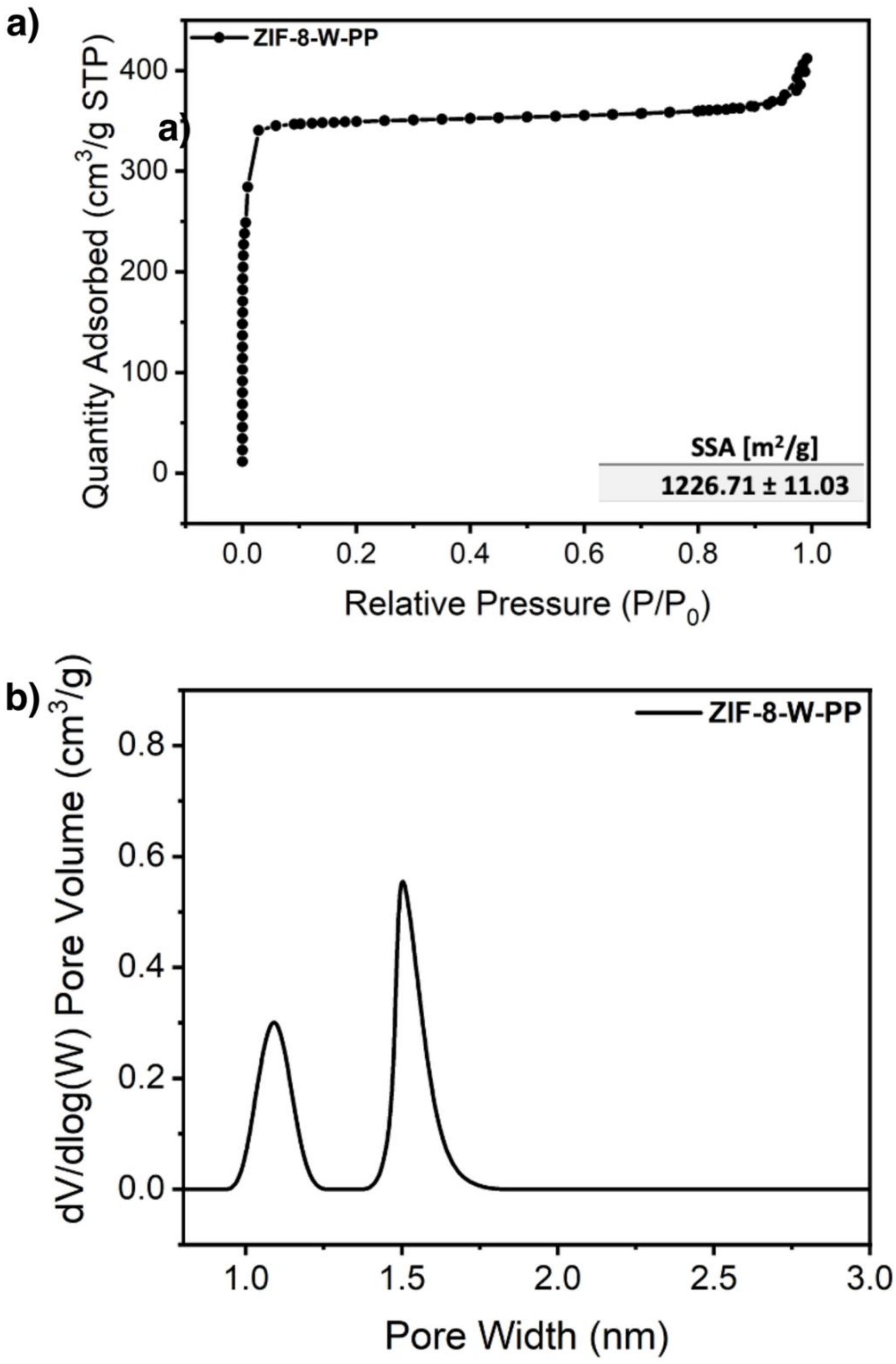

Figure S2. Porous structure parameters of the synthesized ZIF-8 nanoparticles, as determined by: a) N_2 adsorption–desorption isotherms and b) pore size distribution.
